# Experiences of health services and unmet care needs of people with late-stage Parkinson’s in England: A qualitative study

**DOI:** 10.1371/journal.pone.0226916

**Published:** 2019-12-30

**Authors:** Joy Read, Sarah Cable, Charlotte Löfqvist, Susanne Iwarsson, Gergely Bartl, Anette Schrag

**Affiliations:** 1 Department of Clinical and Movement Neurosciences, UCL Queen Square Institute of Neurology, University College London, United Kingdom; 2 Department of Health Sciences, Lund University, Lund, Sweden; Institute of Mental Health, SINGAPORE

## Abstract

**Aim:**

To explore experiences of health services and unmet care needs by people with late-stage Parkinson’s in England.

**Method:**

Ten participants, at Hoehn and Yahr stage 4 or 5, were interviewed using semi-structured open-ended questions. Data were analysed using qualitative thematic analysis.

**Findings:**

Participants reported that whilst under the treatment of specialist hospitals, the majority of care provision had shifted into the community, often because hospital-based services were felt to be difficult to access and have limited benefit to them. When using health-care services, participants frequently experienced having to ‘fit-in’ to service structures that did not always accommodate their complex needs. Despite high levels of disability, participants expressed their desire to maintain their identity, normality of interests and activities in their lives, including remaining in their own homes. This was facilitated by bespoke care and equipment, and positive relationships with care providers. Knowledge on disease management was a key factor in their perceived ability to remain in control. Family caregivers had a central role in facilitating care at home. There was uncertainty about and little planning for the future, and moving to a residential nursing home was perceived an undesirable but potentially necessary option for future care.

**Conclusion:**

Unmet care needs identified by people with late stage Parkinson’s in England include greater flexibility of healthcare structures and bespoke service provision, to accommodate their individual complex needs. Support in their own homes and positive relationships with healthcare providers help People with Parkinson’s (PwP) to maintain a degree of normality and identity, and provision of information help them maintain some control. There is a need for more informed discussions on future care planning for this specific population.

## Introduction

In the UK one in 50 of people over 65 years have Parkinsonism, including Parkinson’s Disease and atypical parkinsonism [1] and this is predicted to double by 2030 [2]. A number of effective pharmacological and non-pharmacological options exist for many of the motor and non-motor problems of Parkinson’s, particularly in the mild to moderate stages of the disease. Nevertheless, people in the late stages experience progressive disability and an increasing number of symptoms that require complex treatment regimens and increasing support from health and social services. Disease progression does not follow a clear trajectory and frequently multi-agency long term provision of healthcare is required to address the complex symptoms that impact on activities of daily life, cause life-limiting disability and reduced quality of life [3, 4]. Many also experience age-related comorbidities in addition to the multiple Parkinson’s symptoms affecting physical functioning, behaviour and cognitive ability. However, knowledge about the lived experiences of people with late stage Parkinson’s is limited.

Medical treatment addresses the management of motor symptoms, such as tremor, postural instability and motor fluctuations [5, 6]; and the many non-motor symptoms that impact quality of life, including psychiatric, cognitive, autonomic, sleep and sensory features [7,8]. However, when significant disability has occurred despite best medical and surgical treatment, the multiple, complex healthcare needs necessitate a greater reliance on health and social care professionals in acute, community, respite and rehabilitation settings, and on support from family members and other informal caregivers. Multidisciplinary, holistic and palliative approaches to complex needs, such as from neurologists, Parkinson’s Disease nurse specialists (PDNS), occupational therapists (OT), physiotherapists, speech and language therapists (SLT) and social workers (SW) are included in international and country-specific guidelines [9, 10]. However, implementation has been shown to be variable and there can be a lack of integrated care [11, 12, 13].

Little is known of the needs of those with Parkinson’s and the care received from their own points of view, particularly in the late stages when disability is greatest and communication is most difficult. This study therefore sought to expand understanding by exploring experiences of service use and unmet care needs of those with late stage Parkinson’s who have high degrees of disability. This information may help address their care needs in the English healthcare system where different healthcare professionals are variably employed in community or secondary care, with some local and regional differences in availability of health and social care.

## Methods

### Sampling and participants

PwP were recruited from the English cohort (n = 123) of the European ‘Care of Late Stage Parkinsonism’ (CLaSP) study, which in six countries aimed to assess the experience of health service use and unmet care needs of those with late stage Parkinson’s using quantitative and qualitative data collection methods [14]. Participants were recruited from both specialist neurologist hospital clinics and from General Practitioner (GP) surgeries. Eligibility included a diagnosis of Parkinson’s according to UK Parkinson’s Disease Society Brain Bank clinical diagnostic criteria [15]; diagnosed for at least seven years, and disease severity stage 4 or 5 during the ‘On’ state on the modified Hoehn and Yahr Scale (H&Y) [16, 17]; or significant disability indicated by a score of 50% or below on the Schwab and England scale [18]. Those with dementia prior to the onset of Parkinsonism, a diagnosis of drug-related “symptomatic Parkinson’s” or unable to clearly communicate were excluded.

For the present study, a purposive sampling approach was used to achieve variation [19] based on existing CLaSP variables of Parkinson’s characteristics, age, gender, residency (including private home and residential care facility; urban and city). Sampling continued until sufficient diversity was reached (n = 12) and as analysis took place alongside the interview process interviews ceased once no new topics emerged. Of those approached to participate only two declined due to personal time pressure, resulting in a final study sample of 10 participants.

Interviews were conducted with 7 men and 3 women over a period of twelve months during 2016. Ages ranged from 70 to 88 and the majority were living in their own homes with a spouse, in urban or suburban areas in and within a 50 mile radius of London, England. On average, participants had had Parkinson’s for 18 years since diagnosis, all were in H&Y stage 4 or 5 and experienced severe mobility restrictions, including being wheelchair dependant or bedbound and therefore reliant on caregivers (Table 1). Varying degrees of neuropsychiatric and cognitive symptoms were evident, as was impairment of voice quality and volume. One participant was communicating via a tablet computer.

**Table 1:** Demographic details and PD characteristics.

		n = 10
Gender	Men–n	7
	Women–n	3
Age	Mean–years	77
	Range–years	70–88
Duration since PD diagnosis	Mean—years duration	18
	Range–years duration	9–28
H&Y stage	Stage 4	5
	Stage 5	5
The unified Parkinson’s disease rating scale(UPDRS) [17]., II (Activities of daily living)	Mean score	26
	Range (where a higher score indicates higher disability (maximum total score = 176))	18–34
UPDRS., III (Motor examination)	Mean score	52
	Range	30–76
Mini-Mental State Examination (MMSE) [20]: used instead of the Montreal Cognitive Assessment (MoCA) to classify Parkinson’s dementia according to the Movement Disorders Society criteria for dementia [21].	Mean score of n = 9 (where a lower score indicates lower cognitive capacity (Maximum score = 30)).	24
	Range	16–29
Residence	Ordinary housing	9
	Residential nursing home	1
Living arrangements	With spouse	5
	With family	2
	Alone	2
	Residential nursing home	1

### Ethics

The study was granted ethical approval from Camden and Kings Cross Research Ethics Committee, London, and was guided by research quality guidelines (Research Governance Framework). PwP were made aware that they did not have to answer any questions they preferred not to, and regular breaks were offered during the interviews, given the frailty of some.

### Procedure

The initial topic guide (Table 2) was developed by members of the CLaSP consortium involved in the qualitative arm of the project. The topics were based on the study objectives and refined during workshops, skype conferences and practice sessions for interviewers led by researchers experienced in qualitative methods, and was used to guide semi-structured interviews. The open-ended interview questions explored the experiences and perceived impact of needs and services, formal and informal support, deficits and barriers to care provision, and future care decisions were explored. For example, “*To what extent are these needs covered*, *and how*?*” “What do you think about the professional care you receive*?*”* Probes were used to seek further in-depth information [22].

**Table 2:** interview topic guide.

Participants living at home	Participants living in residential nursing home
Specific personal needs & meeting of needs Opinion of professional care received Impact on health and life situation Deficits or hindrances to service provision Involvement of family members and friends Reasons for current health choices Opinion about moving to a residential facility/nursing home Anticipated future care needs Provision of information re. future care needs Worries & difficulties (present & future) Pleasures in life	Specific personal needs & meeting of needs Opinion about residential care facilities Decision making process to relocate to institutional setting Feelings around relocating (past & present) Life & support prior to relocation Opinion of professional care received Deficits or hindrances to service provision Involvement of family members and friends Anticipated future care needs Pleasures in life

The timing of the interviews were arranged for a mutually convenient time so that the Parkinson’s medications were at their most effective. After gaining informed consent individual interviews were carried out by one of three authors working in the field of Parkinson’s (JR, SC or GB; two women, one man) whose contact with participants was only through the CLaSP study. All researchers had healthcare or psychology backgrounds, and were further trained for the study by senior researchers experienced in qualitative methodology (SI & CL). Interviews, in the style of a natural conversation, took place at the participant’s place of residence. In the instances where both the person with Parkinson’s and a family caregiver participated, interviews were carried out separately in order to facilitate open discussion (related findings will be reported elsewhere). All interviews, which took an average of 60 to 90 minutes, were recorded using a digital recorder, transcribed verbatim by the interviewers, de-identified and stored securely. Reflective field notes were also collected, typed up and stored securely.

### Analysis

Thematic analysis [23] using an inductive approach, was applied to extract repeated themes using NVivo 11 Pro qualitative software. In brief, transcripts were all read repeatedly by JR and SC to build familiarity with the content and then codes assigned line-by-line to all data of interest. Separate code lists were initially created and then compared face-to-face where overlapping and redundant codes were reviewed to create an agreed joint code list that was applied to analyse further interviews. The recursive process of collating codes under potential themes based on the interrelationships, patterns of meaning and the most significant or frequently occurring was followed by interpretation of the data and the definition of categories and sub-categories. Throughout this process regular communications continued between all authors in order to reflect on the analysis process, and validation of codes, categories and themes.

## Findings

**Fig 1:**
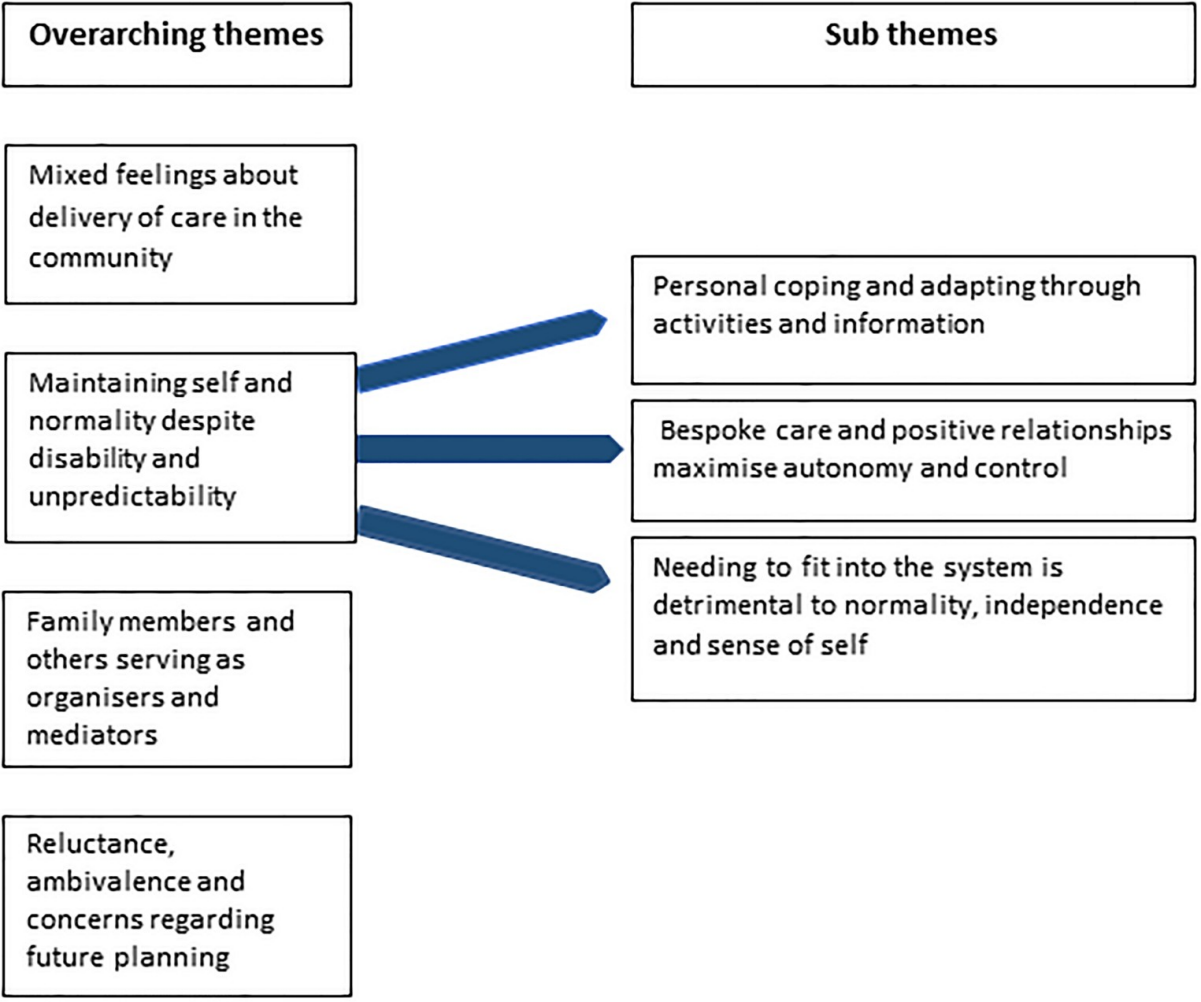
Themes and subthemes.

The analysis revealed four overarching descriptive themes, with three associated subthemes, all of which are presented (Fig 1) and described below.

### Mixed feelings about delivery of care in the community

For all participants interviewed the majority of their healthcare was delivered in the community. Once no longer physically able to attend specialist hospital outpatient clinics some of them described being formally discharged to community services, whereas others just failed to attend as they felt that little more could be done for them and any benefits did not justify the difficulties attending:

*“I can’t get to the hospital*, *because of setting it all up*. *The size of the car*, *couldn’t’ get in the taxi because it had seats*, *where they take the ramps up and sit there*. *Those are very expensive”*.

(Participant: 1010)

*“I know you can’t give*, *you can’t do anything*, *she can’t do anything about it*. *Basically when you have an incurable disease*, *you*, *your*, *the consultant can’t give you the answer.”*

(Participant: 1054)

Once unable to attend hospital outpatients appointments the participants described becoming reliant on their GP and PDNS for management of symptoms, including declining physical ability, fatigue, pain, constipation and urinary tract infections, as well as for the limiting age related comorbidities such as arthritis that several experienced. Regarding the latter, they experienced a lack in coordination and continuity of care for their multiple symptoms and comorbidities, which was seen as a particular challenge given the increasing difficulty of attending multiple appointments:

“*Trying to coordinate doctor*, *doctors*, *nurse*, *neurologist*. *All working on different things*. *A second thing I’d change would be trying to see the same doctor twice*. *All a bit disjointed*. *Like a jigsaw.”*

(Participant: 1094)

As activities of daily life were considerably reduced, the immediate functional needs became the focus for the participants. Home care support increased in quantity and range of tasks for such activities as bathing, dressing and food preparation:

*“I found things have deteriorated very much*. *In the last year things have gone downhill*, *with all the symptoms have got worse*. *Well I couldn’t manage without the carers and er um fortunately I have three good ladies who come morning*, *noon and evening to help me*, *and without their help I couldn’t stay on my own in the flat*. *But with their help I manage*. *Because they do everything*, *they prepare meals*, *cleaning*, *see to the washing* … *I’ve had help for about five years I think and gradually it’s got more you know*, *and I’ve got less able to do things*. *But the last five years things have deteriorated*. *Yes because I used to only have once a day and then it was twice a day*, *now it’s three times because things have got worse.”*

(Participant: 1106)

In addition to reducing physical functioning several participants referred to their unsteadiness and the risk of falling as making them feel unsafe. Falls, for some, resulted in bone fractures and hospital admissions after which increases in support and assistive equipment at home were described, including hoists, bathing aids, kitchen equipment, incontinence aids, all of which facilitated living at home:

*“In the bathroom I’ve got a special seat which also they have to put on a charge the battery*. *And it goes down in the bath and it brings you back up*. *It’s a seat*. *So that’s another thing I’ve got that’s a help*. *Umm I’ve got a commode as well in the bedroom and that’s it*. *Oh and the walker … Well no one likes to have all these things but er you know they’re very useful*. *We’re very lucky that we get them.”*

(Participant: *1106*)

### Maintaining self and normality despite disability and unpredictability

The language of loss was frequently used to describe the continually worsening condition that considerably changed the way the participants lived their lives, and the loss of a future envisaged life. They spoke about their unpredictable physical symptoms and memory problems that led to increased loss of independence, increased reliance on help in their daily lives and considerable restriction in all areas of life. Despite this significant disability and unpredictability of symptoms many spoke of, or demonstrated, their desire to preserve ‘the self’, some normality, an independence and a lifestyle as close to their pre-Parkinson’s selves as was possible. This theme incorporated three subthemes: ‘personal coping and adapting through activities and information’, ‘bespoke care and positive relationships maximise autonomy and control’, and ‘needing to fit into the system is detrimental to normality, independence and sense of self’.

#### Personal coping and adapting though activities and information

Despite the decline in ability and the unpredictability of ON/OFF periods that were described as making it extremely difficult, if not impossible to plan daily activities, or social time with family and friends, interests were maintained where possible, and local links with the community preserved:

*“I used to play bridge a lot*. *Had to stop that now*. *Still play at home once a week*. *I used to play in a couple of clubs as well*. *I’m quite competitive you see.”*

(Participant: *1094*)

Several of the participants at stage 4 described their focus on maintenance of current functioning and prevention of further decline by ensuring a healthy diet and attending singing and exercise classes arranged by local multidisciplinary centres offering Parkinson’s specific facilities and courses, also including information about symptoms, finances and signposting to other relevant services:

*“and there’s dietician*, *yes I’ve had a dietician there*. *And I’ve been back there again recently*, *few months ago*. *The consultant referred me*, *losing weight and I was*, *I was likely to lose weight but they’ve put me on a diet me so I’ve put on another six pounds*. *So I*, *I’ve been steady for the last six months.”*

(Participant: 1038)

As well as increasing support, the use of internal resources were evident with hope, thankfulness about the past, humour, and taking a philosophical view all appearing to placate the sense of loss and need to adapt:

*“No well you’ve got to be sensible you know you’ve got to face facts*, *I suppose like everything when your number’s up your numbers up*! *When it comes to it you just have to go with the wash as they say or go with the crowd*. *What will be will be.”*

(Participant: 1106)

#### Bespoke information and positive relationships maximise autonomy and control

Participants described valuing bespoke information. Knowing as much as possible about their Parkinson’s meant they could discuss concerns in an informed way and maximise autonomy and control:

*“I try to*, *I try to look at the day that’s coming and see what*, *see what I’m gonna have to do*, *like I saw*, *I knew that you were*, *coming so I knew that I had to be*, *I had to turn my pump on at least three quarters of an hour before you came.”*

(Participant: 1054)

Bespoke information was described as being provided by doctors, Parkinson’s support groups, physiotherapists, occupational therapists, with the PDNS being the preferred contact for bespoke information about medication, symptom management and signposting to other services:

*“Sometimes I’ll say to the carers don’t ring the doctors this time*, *ring the nurse*. *And er I said she’s much more knowledgeable about taking*, *um er*, *taking the details and getting it all sorted*. *At the moment we’re trying to get rid of some of the Levopopa [meaning Levodopa]*. *I quite often read about things in the Parkinson’s magazine and I cut it out and show her*. *You know*, *if there is anything that you need*, *information you need you can just write it down and when she comes she is very knowledgeable and she is a good support and er yeah*. *It’s good to know that she’s coming every three months so if you’ve got anything*, *any concerns”*.

(Participant: 1010)

The importance of bespoke approaches to both the content and depth of Parkinson’s specific literature was evident, as was attending support group as in contrast to the positive views above these were also sometimes reported as unhelpful:

*“we have information sent to us about Parkinson’s and my husband reads it*. *Mainly he reads it because I don’t*. *I don’t read it and I don’t*, *I’m not apologizing for that*. *Because umm*, *negative thoughts*, *that may be*, *I feel that if I don’t allow it in*, *then I can stave off the inevitable*. *I reject learning about it because*, *as I say to my husband*, *I said I live it 24 hours a day so umm I don’t want to read about it”*

(Participant: 1064).

Participants described the importance of a positive relationship with care providers and healthcare professionals, who were often referred to as *kind*, *nice*, *knowledgeable*, and *a support*; and with appreciation of someone who could have ‘*a laugh and a joke’*.

One aspect of a positive relationship was described as ‘being understood’ as an individual with particular personality, interests and needs. This was seen to be helped by continuity of care, for example a change in live-in formal carer was described as unsettling until such time as a relationship was formed and personal needs and preferences were understood:

*“She does the breakfast for me*. *Yes*, *nice fresh fruit chopped up*. *She might put up some nice fresh crispies and [carer]s got it just right*! *(…) When they come for a week you’re just getting used to each other and they change over again.”*

(Participant: 1010)

#### Needing to fit into the system is detrimental to normality, independence and sense of self

Many examples were provided of organisational structures shaping the contact between the participants and health professionals and care providers. This included whether GPs carried out home visits and the visit schedules of care providers and supply deliveries:

“*They don’t always turn up*. *And they don’t give you precise times*, *so you’re gonna have to stay here the whole day waiting for somebody to come and deliver something.”*

(Participant: 1059)

Similarly, there were descriptions of having to ‘fit in’ to the busy, inflexible care structures when admitted to hospital. Ward routines did not always accommodate the needs of Parkinson’s, for example, where timing of drug administration was dictated by ward routines and medicines were not understood or given at the correct time:

*“One of the problems was with Levodopa*, *was how you*, *you peak at certain times and drop off the next time and the challenge is to be on an even keel*, *in* [hospital] *they couldn’t read the instructions on the bottle*, *some of them*. *Not sure they gave me the right amount*, *right time.”*

(Participant: 1094)

Such institutional inflexibility was also experienced in care homes where participants experienced that they had to ‘fit in’ to the routine for personal care, meals, drug rounds and even control of room lights or heating:

*“I am woken up at seven for the first lot of tablets*. *The second lot come at nine*. *A carer will come at about ten to start to get me up*, *washed on the loo and dressed*. *So that I might be given breakfast at 11*:*30–12*:*00.”*

(Participant: 1055)

*“I didn’t like the regimentation of it … Not being in your own home*. *Not having*, *not being able to make decisions*. *Not being able to have choices*, *umm.”*

(Participant: 1064)

### Family members and others serving as organisers and mediators

Family members, mainly spouses and children, were described as being the organisers of doctors’ appointments and care at home as well as undertaking household tasks. They were also described as the mediators with healthcare professionals particularly at times of crisis or when a review of deteriorating symptoms was felt to be necessary. Those with more advanced Parkinson’s rarely described initiating the organisation of care themselves instead describing actions and decisions taken by family members. Participants were helped by family members with many health related tasks, either instead of or between the scheduled visits of formal care-workers, including bathing, dressing, and dispensing tablets:

*“She sorts out all my tablets*. *She’s every day*, *she got*, *one for every day*. *And she puts*, *she puts all the tablets in there for me to*, *my supply for each day.”*

(Participant: 1038)

Forgetfulness, feelings of hopelessness, disorientating hallucinations and personality changes were described as disturbing and debilitating features of Parkinson’s, often with considerable impact on those closest:

*“I really feel that I’m living in a false world and there’s nothing to do with me all the time [*referring to hallucinations*]*. *In fact I don’t know what’s real and what’s not nowadays”*.

(Participant: 1071)

*“It’s turned me into a different person*. *Sometimes a very unreasonable*, *umm volatile person*, *who I don’t want to be*. *That’s what Parkinson’s*, *that’s changing me*. *It’s taking the quality of my life and destroying it”*

(Participant: 1064).

The participants appreciated the help of family members and that they enabled ‘normal’ activities such as going shopping:

*“my two daughters come regularly*. *I am very lucky because they live not too far away and sometime if the weather’s alright they put me in the wheelchair and we go shopping and then I can see things*, *and have a bit of fresh air*, *which I*, *I*, *I like*. *Otherwise you can get very bored sitting looking at the four walls you know when you can’t get out*. *But like this they take me and we do a bit of shopping and they come back and provide sometimes meals for me and er they’re very good.”*

(Participant: 1106)

The necessity of handing over tasks to family members that they had previously managed themselves was also described, both positively and negatively, by participants:

*“He does a lot of my paperwork that I can’t do myself*, *so he fills in with the things I can’t do… I’m not allowed to cook now*. *My husband does all the cooking*. *I’m not allowed to do anything*, *and now I rely on my husband and he has his own life to live*, *and he finds he is tending to live my life as well*. *I’m not too good at remembering*. *He does it for me*. *He’s my memory bank*!”

(Participant: 1064)

Non-family members, such as neighbours or help found through support groups or charities, including Age Concern, also helped with tasks such as cleaning and gardening which meant participants could remain at home:

*“they help the*, *somebody to cut the trees and do the garbage*, *cleaning up for us*, *what we couldn’t do ourselves*, *yeah*. *They were*, *they were helpful*, *very helpful there.”*

(Participant: 1038).

### Reluctance, ambivalence and concerns regarding future planning

Although not necessarily overtly told that ‘nothing more can be done’, several participants perceived that to be the case, reportedly after completing physiotherapy courses without physical improvement, lack of symptom improvement despite medication changes, and reduced involvement with health professionals due to limited availability of PDNS and OT’s, and GPs unable to do home visits:

*“and er I begin to think well ‘what’s the point*?*’ because I stay on the same medication*. *So long*, *and nothing’s changed*. *Well I think there’s just not anything else they can do*. *It’s just a question of how long I’m going to be here for and how am I going to cope*. *And that’s it really*. *I don’t think there is anything anyone can do.”*

(Participant: 1106)

Despite this, there was an obvious ambivalence and reluctance to discuss the future, end of life or palliative care, with participants either avoiding the question with long silences, contradictory statements or unrelated responses, or with honesty:

*“Very*, *very difficult*, *very difficult to think about the future.”*

(Participant: 1054)

Instead of discussing the future, participants residing at home focused on their current care and spoke about remaining in their own homes for as long as possible. When probed there was evidence that participants had concerns about the future and finding appropriate living arrangements. There were also concerns about the ability to cope with the uncertainty of what lay ahead including the costs of future care.

When the long term future was actually discussed, participants spoke about moving into residential nursing homes. Perceptions of these facilities were generally negative, with references to media reports of bad practice, the negative experiences of parents or family members, or their own short term admissions for respite or following hospital discharge. Such a move was also related to the concerns of being separated from their spouses, the loss of independence, and being under stimulated and isolated from friends and family. Despite wishing to remain at home participants spoke with resignation that this might be inevitable, particularly out of consideration for other family members and from not wishing to be a burden:

*“Well without him I wouldn’t be*, *I would have to go into a care home I should imagine.”*

(Participant: 1064)

*“If things get much worse*, *that*, *you know I’ll have to say yes*. *I’ll have to go into a home*, *because it’s not fair on them*. *Well if I have to I have to*. *I won’t have a choice you know because they’ll say “no mum*, *you know*. *It’s too much of a worry for us now you are on your own*. *At least we know you’re in the place with other people all the time”*. *Which is true.”*

(Participant: 1106)

## Discussion

This study expands the literature by reporting findings from the experiences, views and opinions of health services and care needs specifically of individuals at the late stage of Parkinson’s, including those with severe mobility restrictions and one participant communicating via a tablet computer. Such participant experiences have received little attention in the literature and there are no comparable studies. The findings provide insight into their views and show that their perceived needs are only partially met by the current structures of health and social care provision in England.

It is essential to understand the lived experiences and service use of those in the later stages of Parkinson’s given the high disability when progression to this stage has occurred, the increasing prevalence of Parkinson’s with aging of the population, and the increasing direct and indirect impact on those with Parkinson’s, on their family caregivers and the healthcare system and social services [24, 25].

Participants with late stage Parkinson’s in this study described the multiple, fluctuating and unpredictable symptoms that increasingly affect and restrict all areas of life. Despite a dynamic unremitting cycle of loss and the stepwise decline in ability there is an evident desire to hold on to a sense of the self, individual identity and activities despite their lives becoming perceptively smaller with increasing reliance on informal and formal caregivers. The desire to maintain ‘normalcy’ has previously been found in earlier disease stages [26] and it is notable that it remains despite significant disease advancement. Similarly, the desire to maintain the individual person has been reported as important for carers of those with dementia [27] and in the re-engagement process for long term stroke survivors [28].

The care needs of participants largely centre on a desire to remain at home, with needs predominantly met in the community. It is at this point the PDNS, who are often accessible by phone and carry out home-visits, seem to be of significant importance. Particularly for the provision of bespoke information, symptom management, and emotional support; many of which are elements acknowledged as important in other neurological conditions also [29]. As many PDNS are hospital based they also provide an important link to specialist neurologists and hospital services, of importance as it has been shown that satisfaction in care is more likely when under a specialist neurologist than a generalist [30]. PDNS are in the position to personalise care by exploring the symptoms seen as paramount importance to the person with Parkinson’s. Similarly can gauge the appropriate type and depth of information wanted, and can accommodate personal coping strategies, and the ambivalence and reluctance to discuss prognosis and the future as found in this cohort and other studies [31].

With disease progression, focus becomes increasing on the maintenance of daily activities and immediate functional needs. Much essential support for these areas comes from family members who frequently organise formal care, facilitate normal social activities and household life, provide care and fill gaps in service provision; which often requires the participants to relinquish previous roles, which was described as not always being an easy transition. Many tasks that could not be met through formal statutory services are frequently undertaken by family members, which enable participants to safely remain at home and maintain a good quality of life. These findings highlight the importance of supporting informal caregivers, for example, with multicomponent support programs [32], not only to allow PwP to retain a sense of self and to improve their quality of life and daily functioning, but also to enable them to stay at home rather than move to a residential nursing home, which was generally viewed as undesirable and is costly to society.

In terms of care provision, positive relationships with the breadth of recommended health professionals (9,10), and care givers are an important aspect for participants. In addition to the quality of relationships, ‘being understood’ and receiving bespoke information, equipment and care also shaped perception of service provision and mediated adjustment. The importance of positive inter-relationships in care provision, person-centred specialist care and information have been reported in earlier stages of Parkinson’s [33, 34, 35], and is shown to be undiminished by disease stage in these participants.

Whilst generally appreciative of the support and services received from individual providers, as in previous studies [11,12,13] participants spoke of variability of service provision. They also spoke of a lack of co-ordination and lack of continuity of treatment and care, particularly during hospital admissions, which underlines the importance of initiatives that develop local processes to ensure best possible outcomes from a hospital admission [36]. In addition, whilst there was an increasing need and reliance on services, participants often felt they had to fit into busy inflexible service structures that did not always accommodate the increasing decline in ability, and multiple complex symptoms of late stage Parkinson’s, comorbidities and ageing. Perhaps it is therefore unsurprising that personalised care is within the top five key research priorities for those interviewed by Parkinson’s UK [37].

The transition from hospital to community services is often led by the physical inability to attend appointments rather than a clear plan of care, and at this stage many participants felt resigned that no more could be done to improve their situation. The move from hospital to community services is complicated in England by the funding division between social care and healthcare provision and the GP as the “gatekeeper” initiating referrals to other health care professionals depending on availability and local or national funding. This difficulty may in the future be supported by emerging new technologies not yet used by this cohort [38].

Similarly, current structures of care do not appear to be conducive to, or encourage, planning long term future care or of a planned change from proactive treatment of symptoms and complications to palliation and finding better ways of managing and living with deterioration. This is despite an increased awareness of the potential benefit of palliative care interventions in Neurodegenerative Diseases [9,10, 39] and such approached being evident in other conditions including cancer [40], dementia [41] and other neurological conditions such as Motor Neuron Disease [11]. Also there was no evidence of advanced care planning, about which there are misconceptions and no clear best practice methods in Parkinson’s [42]

It is noteworthy that alongside a lack of knowledge about palliative care as reported elsewhere [43] and a general reluctance to look at future care needs there was an acceptance that long-term institutional care would be necessary with significant disease advancement, although seen as the last resort. Evidence however suggests that frequently those with neurologic conditions with palliative care needs are regularly hospitalized at the end-of-life [44,45].

### Study limitations and strengths

The sample was small, but consistent with sample sizes in qualitative research and no additional topics were noted during the last interviews. Consistent with the overrepresentation of men amongst PwP [1], there were more men than women in our sample. Whilst sampling occurred from a defined geographical area in and within a fifty mile radius of London, and therefore possibly not representative of the whole of England, the urban and suburban settings were considered suitably diverse to give an informative picture of residence and experience of service use. However, there was a lack of ethnic diversity limiting the generalisability of these findings. Similarly, participants were recruited from both specialist neurologist hospital clinics and from GP surgeries, offering some diversity, however different experiences might have been reported by recruits from geriatric-led and other general clinics.

Several participants were dependent on family and formal care providers and may therefore have felt uncomfortable criticising them and so findings from other samples may differ. Moreover, due to dysarthria and neuropsychiatric symptoms it was not always possible to capture the voices of participants at stage 5 with the most severe symptoms, although it was possible to include one participant communicating via a tablet computer. Similarly, concentration, memory changes and hallucinations could have had an impact on perception and therefore on the information given. Despite a multifaceted recruitment strategies it was only possible to include one participant residing in a nursing home. However, several participants had previously spent time in such institutions, often after hospital discharge, and their viewpoints are reflected.

However, the study was based on diversity sampling using descriptive information from a well-defined cohort. The team of authors represents clinical and scholarly expertise including neurology, nursing, occupational therapy, gerontology and psychology not only from England but also from Sweden. Following rigorous methodology, this interdisciplinary and cross-national constellation of researchers collaborated in an iterative process of critical appraisal during the entire process, which served well to ensure the validity and trustworthiness of findings.

### Implications

This study has several implications for clinical practice and community care for this very vulnerable group who have complex and unpredictable needs. The best service provision was described as being collaborative between healthcare providers, being individually tailored to address the heterogeneous and complex nature of Parkinson’s in different individuals, and with continuity of care to enable a personal relationship that supports preservation of dignity and a feeling of self. PDNS were particularly helpful, by providing specialised information and advice in the context of the individual’s own circumstances, and providing a liaison between agencies.

Care for those with Parkinson’s initially requires a focus on diagnosis, the communication of which is relevant many years after the initial diagnosis. Focus then moves to the management and control of the variable and changing clinical features during the disease course. There is then a particular unmet need to address the transition from specialist clinic based care to community care in the more advanced stages when symptom management becomes less effective and planning end of life care needs to be included. This could be addressed through the introduction of a clear plan of care, and a personalised care plan or disease-specific patient-centred care pathway, including palliative care, to facilitate a more cohesive, person-centred and effective management of care. This should include discussion of long-term future care and advanced care planning in a timely manner. This requires greater and earlier accessibility of palliative care services to this group as well as more integrated Parkinson’s specialist, community and palliative services and training of PDNS and other Parkinson’s specialists in the advanced care planning.

One of the key challenges identified was the difficulty of fixed generic healthcare structures to address the management complexities of Parkinson’s, particularly in non-specialised settings. Adapting care and service provision to the variable and unpredictable nature of Parkinson’s through more flexible appointments and a responsive system, and integrated healthcare could address some of these.

The findings also highlight the importance of providing appropriate community based emotional and practical support for family caregivers as many of them fill gaps in service provision and are often the main mediators between those with Parkinson’s and services to make sustained living at home possible.

## Conclusion

Late stage Parkinson’s leads to profound disability affecting all areas of life, and is shaped not only by the manifestations of the disease but also the services and care received formally and informally. Patients facing increasing levels of disability due to their condition could be better supported by improved availability of specialist, bespoke, home-based services, earlier access to palliative care options, person-centred decision-making, better coordination across health and social care service providers, and more support available for family caregivers.
